# The promises and perils of artificial intelligence in clinical medicine: a dual perspective from internal medicine and pediatrics

**DOI:** 10.3325/cmj.2025.66.243

**Published:** 2025-08

**Authors:** Lovro Lamot, Robert Likić

**Affiliations:** 1 University of Zagreb School of Medicine, Zagreb, Croatia; 2 Department of Pediatrics, Division of Nephrology, Dialysis and Transplantation, University Hospital Centre Zagreb, Zagreb, Croatia; 3 Department of Internal Medicine, Division of Clinical Pharmacology, University Hospital Centre Zagreb, Zagreb, Croatia; *robert.likic@mef.hr*

The integration of artificial intelligence (AI) into clinical medicine represents one of the most profound paradigm shifts in the history of health care. Its proponents emphasize AI's capacity to process and synthesize vast, heterogeneous clinical data, offering support for clinical decision-making, enhancing pharmacotherapeutic precision, and even accelerating translational research (1-3). At the same time, many clinicians remain appropriately cautious, recognizing AI’s current limitations, its potential for error propagation, and the risk of undermining clinical judgment if not properly governed (4,5). While much of the published debate remains speculative or theoretical, real-world clinical practice is increasingly intersecting with AI applications. This article offers a joint perspective from two actively practicing physicians, one in internal medicine and clinical pharmacology and the other in pediatrics, each drawing directly from personal experience with AI-powered clinical support systems such as ChatGPT-4o.

In the field of internal medicine and clinical pharmacology, the potential for AI to transform daily practice is already becoming reality. A clear example is its role in managing complex polypharmacy and suspected adverse drug reactions, where clinical pharmacologists often confront highly intricate medication lists involving both prescription drugs and over-the-counter supplements (6). One case involved a 74-year-old woman presenting with chronic generalized pruritus persisting for over two years. She had multiple comorbidities, including Hashimoto’s thyroiditis, non-insulin-dependent diabetes mellitus, hypertension, and chronic constipation. Her medication list exceeded fifteen agents, encompassing both pharmacologic treatments and numerous supplements such as dehydroepiandrosterone, hydrocortisone, omega-3 fatty acids, zinc, magnesium, vitamin B complex, coenzyme Q10, and phytoestrogens. Traditional medication reconciliation in such cases is time-consuming, and the identification of potential delayed hypersensitivity reactions or iatrogenic contributors to her symptoms requires integration of multiple pharmacological domains.

By leveraging ChatGPT-4o, a comprehensive medication assessment was completed within minutes. AI assistance rapidly synthesized pharmacokinetic pathways, cross-reactivity profiles, and rare hypersensitivity reports, while also proposing a rational protocol for systematic withdrawal and challenge to elucidate potential offending agents (7). The system rapidly consulted pharmacovigilance databases and cross-referenced current literature, generating a depth of analysis that would traditionally require several hours or days. Importantly, while AI provided exhaustive and up-to-date suggestions, clinical oversight remained indispensable in prioritizing drug discontinuation order and closely monitoring patient response. Nevertheless, the efficiency and breadth of AI-supported analysis fundamentally elevated the clinician’s capacity to navigate such complex pharmacotherapeutic scenarios.

AI also proved transformative in situations of complex differential diagnosis, as illustrated in the management of a critically ill adult woman presenting with multi-organ failure, requiring mechanical ventilation, intermittent hemodialysis, antifungal therapy for candidiasis, and intensive supportive care. The central diagnostic dilemma revolved around distinguishing between multisystem inflammatory syndrome in adults (MIS-A) and systemic lupus erythematosus, as both entities present with overlapping inflammatory, cardiac, renal, hepatic, and hematological involvement (8). Through iterative consultations, the AI-assisted system generated differential diagnostic frameworks, identified gaps in the existing laboratory workup, and proposed organ-specific therapeutic interventions (9,10). In this context, AI served as a cognitive amplifier for the multidisciplinary team, providing access to rapidly evolving literature on MIS-A phenotypes, recent case series, and evidence-based immunosuppressive strategies. Furthermore, AI-assisted pharmacology modules provided drug-drug interaction assessments, suggesting alternative nephroprotective strategies to minimize further renal injury when considering diuretic therapy modifications such as replacing furosemide. Once again, human expertise remained central, but the AI-powered system significantly expanded the diagnostic bandwidth and improved therapeutic precision in real time.

Perhaps one of the most powerful examples of AI's emerging role lies in the application of pharmacogenomics to guide personalized therapy. In a case involving a 16.5-year-old girl suffering from chronic anxiety and nausea, complicated by poor tolerability to multiple prior psychotropic regimens, pharmacogenomic profiling revealed actionable variants in *CYP2C19* and *CYP2D6*. These findings raised critical concerns regarding the metabolism of several first-line selective serotonin reuptake inhibitors (11). The AI system rapidly integrated the guidelines of Clinical Pharmacogenetics Implementation Consortium, Food and Drug Administration labeling, developmental pharmacokinetics, and recent pharmacogenomic literature to generate a ranked list of viable antidepressant options with individualized dosing recommendations (12,13). Furthermore, it allowed dynamic modeling of potential serotonergic side effects based on predicted plasma concentrations. This capacity for an individualized approach in the context of adolescent psychiatric therapy would be extraordinarily difficult to achieve without AI-assisted computational analysis.

Beyond individual patient care, AI is having an increasingly helpful role in medical education, research design, and wound care management. So far, it has been used to create comprehensive multiple-choice question banks for student assessments, to build interactive, case-based teaching scenarios, and to support *in silico* drug repurposing efforts (14). In wound care, AI-driven visual analysis has also contributed by tracking wound healing over time through serial photograph analysis, thereby offering additional insights that can aid clinicians in tailoring treatment protocols according to the wound healing progress (15).

Still, despite these promising developments, experiences in pediatric medicine continue to highlight important reasons for caution. The complexity of pediatric care, where developmental stages and weight-based variations introduce significant variability, poses particular challenges for AI systems (16). In routine pediatric renal ultrasound assessments, for instance, AI has been able to quickly measure parameters such as renal length, cortical thickness, and parenchymal echogenicity. However, several reports have shown that AI sometimes struggles to distinguish between harmless developmental variations and true pathology, occasionally flagging normal findings as potentially abnormal (17). Although these discrepancies may seem minor, they carry the risk of prompting unnecessary tests or interventions if the AI’s suggestions are followed without careful human oversight.

A particularly instructive case underscoring the current limitations of AI in pediatrics involved a 13-year-old girl presenting with persistent fatigue, low-grade fever, newly diagnosed hypertension, elevated erythrocyte sedimentation rate and C-reactive protein, a non-specific rash, and an unremarkable immunologic screen including negative antinuclear antibodies and antineutrophil cytoplasmic antibodies, and normal complement levels. Notably, plasma renin activity was elevated while aldosterone remained within reference range, a constellation that initially raised suspicion for secondary hypertension due to renal or renovascular pathology. Subsequent imaging, including Doppler ultrasonography and CT angiography, failed to reveal any structural or vascular abnormalities. When entered into the AI system, the differential diagnosis was dominated by endocrine causes, renal parenchymal disease, and infectious etiologies. Despite the presence of systemic inflammation and unexplained hypertension, vasculitis was not included among the proposed options. Even after expanding the clinical data set with elevated renin, imaging findings, and detailed symptom chronology, AI systems failed to surface vasculitis as a plausible consideration. This omission likely stemmed from a mechanistic overreliance on classification criteria, which typically include immunologic serologies, specific imaging findings, or biopsy-confirmed vascular inflammation. In reality, the most plausible diagnosis was only clinically entertained after further evolution of symptoms and persistence of diagnostic ambiguity. The AI system’s failure to recognize this entity illustrates a deeper limitation: a lack of clinical anticipation. As noted in recent literature, many AI systems still function as uninterpretable “black boxes,” which makes it challenging to explain or validate their diagnostic reasoning or omissions (18). Unlike experienced pediatric subspecialists who intuitively weigh evolving symptom trajectories and remain alert to partial or incomplete phenotypes, current AI models are structurally incapable of forming provisional hypotheses in the absence of fully realized diagnostic features. This case exemplifies a broader concern: in rare pediatric immunologically mediated diseases, especially those lacking standardized guidelines or widely validated diagnostic pathways, AI tools may underperform or even mislead if not critically supervised. The failure was not due to lack of data processing but due to a fundamental deficit in interpretive foresight, the type of clinical reasoning that allows for dynamic hypothesis generation, pattern recognition in evolution, and judgment under uncertainty. Until AI systems can incorporate probabilistic reasoning across time and uncertainty, particularly in children where phenotypes are fluid and diagnostic criteria underdeveloped, their role must remain strictly supportive, never substitutive.

Even in relatively straightforward tasks in pediatric nephrology, such as unit conversions and reference value calculations for metabolic urine analysis, AI tools can yield inconsistent outputs. Such calculations are based on established formulas and reference standards that include factors like body height, weight, body surface area, 24-hour urine volume, creatinine excretion, and concentrations of various solutes. While AI systems should simplify these computations and reduce the time needed for patient management, the outputs frequently contain mistakes due to incorrect application of age-specific norms, faulty unit conversions, or failure to properly adjust for body size. Therefore, these issues often require physicians to review and manually correct the results to ensure patient safety and best health outcomes. This exposes a key limitation of the current AI systems: mistakes can occur unless the system is specifically adapted to the detailed requirements of pediatric practice.

Furthermore, AI-generated advice on medication dosing in pediatric patients has so far demonstrated several shortcomings. For instance, algorithms that calculate doses based on body weight have occasionally failed to adjust for organ changes with age, particularly in premature infants, newborns, or children with specific syndromes (19). This becomes even more important when prescribing drugs with a narrow therapeutic index, where even slight miscalculations in dosing can lead to significant clinical risks. Another important issue is that AI systems often communicate their outputs with a tone of authority, regardless of accuracy. Such confident, yet incorrect, suggestions can lead to automation bias, increasing the odds of clinicians trusting the AI’s output without critical review (20). This risk becomes even more pronounced in high-pressure clinical situations, where rapid decisions are often needed (21).

Taken together, these experiences emphasize the central point of our joint statement. While AI brings enormous potential to clinical medicine, it cannot yet substitute for the expert judgment of trained physicians. The more prudent path is to build hybrid models where AI supports and strengthens, rather than replaces, clinical expertise. For safe implementation, there will need to be clear audit mechanisms, transparent validation processes, AI systems tailored to specific specialties, and comprehensive training programs to equip physicians with the necessary AI literacy (22).

In the hands of properly trained clinicians, AI can indeed revolutionize clinical practice by enhancing diagnostic accuracy, personalizing therapy, and accelerating research translation. However, clinicians must remain vigilant stewards of patient safety, critically appraising AI recommendations while using their domain expertise to contextualize machine-generated outputs. The future of AI in medicine is neither utopian nor dystopian. It is hybrid, collaborative, and above all, accountable.
